# R&D on glass fiber reinforced epoxy resin composites for superconducting Tokamak

**DOI:** 10.1186/s40064-016-2995-6

**Published:** 2016-09-15

**Authors:** Nannan Hu, Ke Wang, Hongming Ma, Wanjiang Pan, Qingqing Chen

**Affiliations:** 1 Power Research Institute of Yunnan Power Grid Co., Ltd., Kunming, 650217 China; 2 Institute of Plasma Physics, Chinese Academy of Sciences, Hefei, 230031 China; 3 Yin Sudan Electric Co., Ltd., Hefei, 230031 China

**Keywords:** Cryogenic temperature, Glass fiber reinforced epoxy resin composites, Superconducting Tokamak

## Abstract

The glass fiber reinforced epoxy resin composites play an important role in superconducting Tokamak, which are used to insulate the metal components, such as superconducting winding, cooling pipes, metal electrodes and so on. For the components made of metal and glass fiber reinforced epoxy resin composites, thermal shrinkage leads to non-ignorable thermal stress, therefore, much attention should be paid on the thermal shrinkage rate of glass fiber reinforced epoxy resin composites. The structural design of glass fiber reinforced epoxy resin composites should aim at reducing thermal stress. In this paper, the density, glass fiber content and thermal shrinkage rate of five insulation tubes were tested. The testing results will be applied in structural design and mechanical analysis of isolators for superconducting Tokamak.

## Background

In superconducting Tokamak, cryogenic temperature insulating materials were widely used in super-conducting magnets system, such as superconducting magnets, high temperature superconducting current leads and feeders (Canfer et al. 2011, 2013; Bondarenko et al. 2009; Hemmi et al. 2009; Usami et al. 1999; Li et al. 2014; Ivanov et al. 2012; Humer et al. 2013; Glukhikh et al. 2000; Bursikov et al. 2014). Compared with insulating materials used in room temperature, the cryogenic temperature resistant insulating materials were fabricated in room temperature but used in cryogenic temperature, such as 77, 4.2, 1.8 K and so on. Due to different thermal shrinkage rate of metal and insulating material, thermal stress will be come into being during cool-down from room temperature to cryogenic temperature. As a result, excellent mechanical properties at cryogenic temperature of the cryogenic temperature resistant insulating materials are expected (Usami et al. 1999). To reveal the influence of content and glass fiber direction on the performance of glass fiber reinforced epoxy resin composites, R&D on glass fiber reinforced epoxy resin composites for superconducting Tokamak was performed.

## Material design (Sawa et al. 1995; Schutz 1998)

### Epoxy resin system

**Fig. 1:**
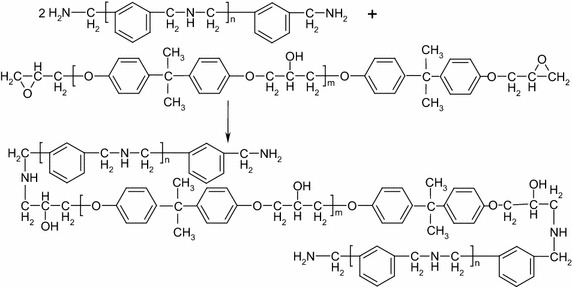
Curing of DWZ epoxy resin system

To resist crack propagation, DWZ cryogenic epoxy resin system was developed for glass fiber adhesive, which includes two components. Component A is a mixture of bisphenol-A epoxy resin, Qishi toughening agent and silane-coupling agents (KH-560), and component B is aromatic condensation amine (GY-051) polymer, the mass ratio of component A and component B is 4:1. The curing chemical reaction of the two components is as shown in Fig. 1, which is a bimolecular chemical reaction. The principal of chemical reaction is as follows:

- The combination of oxygen atom of epoxy groups and the hydroxyl hydrogen atom of aromatic condensation amine (GY-051) forms hydrogen bonds.
- Hydrogen bonds leads to further polarization of epoxy group, which results in the nucleophilic attack of C atom of epoxy groups by N atom of amino-group. Therefore, each epoxy group will be opened by one active hydrogen from amino-group. As a result, the cured epoxy resin system is intermolecular cross-linking.

**Fig. 2:**
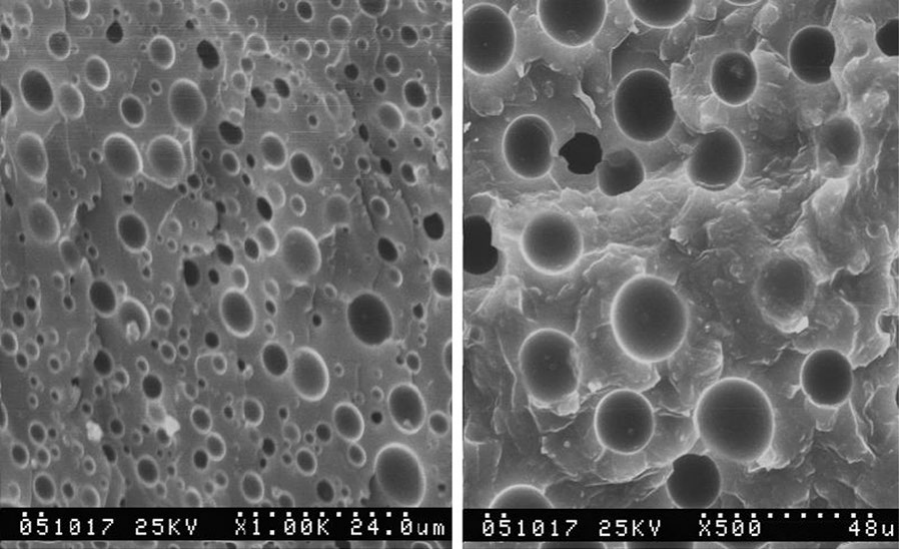
The scanning electron microscopy from the fracture area of pure DWZ epoxy resin at 293, 77 K from *left* to *right*

In the cured DWZ cryogenic epoxy resin system, epoxy resin is continuous phase and curing agent is dispersed phase, the toughening effect of the two-phase structure under cryogenic temperature is effective, which corresponds to insensitivity service temperature. Table 1 shows the tensile strength of DWZ epoxy resin at different temperature. Table 2 shows the shearing strength testing results of DWZ epoxy resin at different temperature. Figure 2 shows the scanning electron microscopy from the fracture area of pure DWZ epoxy resin at 293 and 77 K.

**Table 1:** Tensile strength testing results of DWZ epoxy resin at different temperature

Specimen name	Room temperature (293 K)				Liquid nitrogen temperature (77 K)			
	No.	Tensile strength (MPa)	Average value	Standard deviation	No.	Tensile strength (MPa)	Average value	Standard deviation
DWZ	1	63.40	59.79	3.23	4	75.81	84.47	9.99
	2	57.15			5	95.40		
	3	58.83			6	82.21

**Table 2:** Shearing strength testing results of DWZ epoxy resin at different temperature

Specimen name	Room temperature (293 K)				Liquid nitrogen temperature (77 K)			
	No.	Shearing strength (MPa)	Average value	Standard deviation	No.	Shearing strength (MPa)	Average value	Standard deviation
DWZ	1	10.72	9.38	1.43	5	13.63	13.13	0.50
	2	9.92			6	12.51		
	3	7.37			7	12.97		
	4	9.51			8	13.42

Due to different modulus of elasticity of continuous phase and dispersed phase, forces can be detoured and transmitted along the interface between the two phases, so the stresses can be consumed around the border of spheroidal structure, especially the thermal stresses. More energy was consumed due to the deformation of continuous phase at the interface and the brittle rupture of particles, so the stress concentration was dispersed, the crack propagation was prevented, stress state was improved and the sensitivity of mechanical property to temperature was reduced. Because the participation of silane-coupling agents, better linkage at the interface of the two phases was obtained. Therefore, the toughness of DWZ epoxy resin system under cryogenic temperature was increased. In addition, the mechanical property degradation due to dispersed phase softening with increasing temperature was improved. Clearly, the mechanical properties of DWZ cryogenic epoxy resin system under room temperature and cryogenic temperature are excellent. Therefore, the DWZ epoxy resin system can fulfil expected requirements.

### Glass fiber

**Fig. 3:**
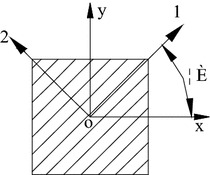
Off-axis model of glass fiber reinforced epoxy resin composites

E and R glass fibers are compared below. Tables 3 and 4 show the chemical composition of E and R glass fibers respectively. Table 5 shows the specifications of these two types of glass fiber. Due to no Boron, R glass fiber can be used in superconducting Tokamak involving radiation. In Fig. 3, xoy is natural coordinate system, when the directions of natural coordinate axes (x and y) are identical with the directions of the principal axes (1 and 2) of the glass fiber reinforced epoxy resin composites, the modulus of elasticity parallel to the direction of fiber glass and vertical to the direction of fiber glass can be calculated based on the rule of mixture of composite, as shown in formulas (1) and (2) respectively.

1 $$E_{1} = E_{f} V_{f} + E_{m} (1 - V_{f} )$$

2 $$\frac{1}{{E_{2} }} = \frac{{V_{f} }}{{E_{f} }} + \frac{{1 - V_{f} }}{{E_{m} }}$$

**Table 3:** Chemical composition of E glass fiber

E type glass fiber	SiO_2_	Al_2_O_3_	CaO	Mg0	B_2_O_3_	K_2_O + Na_2_O
Content (%)	52–55	13–25	15–17	3–5	7–9	<0.8

**Table 4:** Chemical composition of R glass fiber

R type glass fiber	SiO_2_	Al_2_O_3_	CaO	Mg0	BaO
Content (%)	50–55	20–25	10–15	10–15	1–5

**Table 5:** The specifications of glass fiber

Specification	Virgin fiber tensile strength (MPa)	Modulus of elasticity (GPa)	Density (g/cm^3^)	Elongation (%)	Impregnated strand tensile strength (MPa)	Softening point temperature (°C)
E-Glass	3140	73	2.54	4.8	1860	850
R-Glass	3000–3400	80–83	2.65	–	–	895

In formulas (1) and (2): E_1_—the modulus of elasticity of composite parallel to the direction of glass fiber; E_2_—the modulus of elasticity of composite vertical to the direction of glass fiber; E_f_—the modulus of elasticity of glass fiber; E_m_—the modulus of elasticity of epoxy resin; V_f_—the volume percent of glass fiber in composite.

Obviously, E_1_ and E_2_ were mainly determined by E_f_, E_m_ and V_f_. Actually, glass fibers in composite are wound at an angle of θ to the vertical axis, the off-axis model of glass fiber reinforced epoxy resin composites is as shown in Fig. 3, E_x_ and E_y_ can be calculated with formulas (3) and (4) respectively. Obviously, E_x_ and E_y_ can be changed by adjusting the angle of θ when E_f_, E_m_ and V_f_ are known.

In formulas (3) and (4): µ_12_—the Poisson’s ratio of composite in 1–2 plane; G_12_—the shear modulus of elasticity of composite in 1–2 plane

3 $$\begin{aligned} \frac{1}{{E_{x} }} & = \frac{1}{{E_{1} }}\cos^{4} \theta + \left( {\frac{1}{{G_{12} }} - \frac{{2\mu_{12} }}{{E_{1} }}} \right)\sin^{2} \theta \cos^{2} \theta \\ & \quad + \frac{1}{{E_{2} }}\sin^{4} \theta \\ \end{aligned}$$

4 $$\begin{aligned} \frac{1}{{E_{y} }} & = \frac{1}{{E_{1} }}\sin^{4} \theta + \left(\frac{1}{{G_{12} }} - \frac{{2\mu_{12} }}{{E_{1} }}\right)\sin^{2} \theta \cos^{2} \theta \\ & \quad + \frac{1}{{E_{2} }}\cos^{4} \theta \\ \end{aligned}$$

In term of mechanical properties, the modulus of elasticity of R glass fiber is higher than E glass fiber, which means R glass fiber reinforced DWZ epoxy resin composites is more sensitive to stress concentration than E glass fiber reinforced DWZ epoxy resin composites.

### Glass fiber reinforced epoxy resin composites

In superconducting Tokamak, glass fiber reinforced epoxy resin composites can be used to develop isolators, which were used to convey coolant and insulate the cooling pipes in superconducting magnets system. However, manufacturing process has great effect on the properties of glass fiber reinforced epoxy resin composites. To reveal the influence of different winding process parameters on the density, void ratio and thermal shrinkage rate of glass fiber reinforced epoxy resin composites, five insulation tube specimens were manufactured with R *g*lass fiber reinforced DWZ epoxy resin composites involving different winding process parameters, such as glass fiber pattern, glass fiber geometry, glass fiber angle, curing temperature and curing time.

**Fig. 4:**
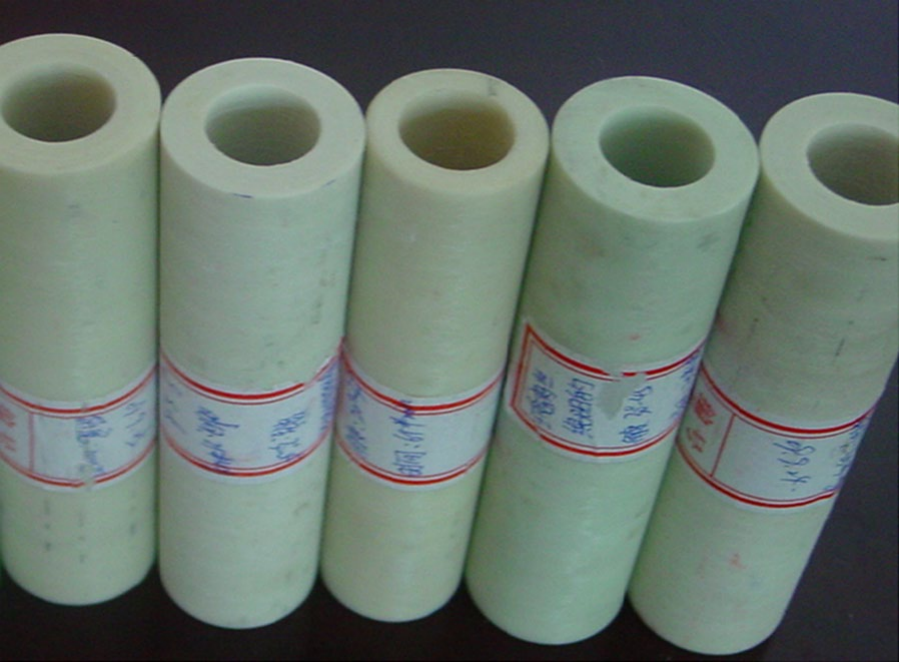
Insulation tube specimens

Glass fiber filament and glass fiber tape with different geometry and different fiber angle were used to wind the insulation tube specimens. The glass fiber tape was woven by using glass fiber filament. The insulation tube specimens are as shown in Fig. 4, the winding process parameters are as shown in Table 6.

**Table 6:** Winding process parameters of insulation tube specimens

Insulation type	Glass fiber	Geometry (mm)	Fiber angle (°)	Curing temperature (°C)	Curing time (h)
L1	Filament01	500 tex	30–45	100	2
L2	Filament02	250 tex	20–30	100	2
L3	Tape	w = 20, d = 0.1	30–45	100	2
L4	Filament01	500 tex	30–40	100	2
L5	Filament02	250 tex	30–40	100	2

## Tests

### Density test principia and methods

On the structure, the glass fiber reinforced epoxy resin composite consists of DWZ epoxy resin system and R glass fiber, according to GB/T 1463-2005, the density of R glass fiber reinforced DWZ epoxy resin composite can be tested by using buoyancy method. Formula (5) was used to calculate the density $\rho_{c}$ of specimens. Mass of specimens was measured in air, and the volume of specimens was converted by buoyancy and the density of water. To obtain the accurate value of buoyancy, the specimen was dipped into the water and suspended from the upper rim of a cup.

5 $$\rho_{c} = \frac{{M_{1} }}{V} = \frac{{M{}_{1}}}{{M_{1} - M_{2} }}\rho_{w}$$

In formula (5), M_1_: mass of specimens in air; V: volume of specimens; M_2_: mass of specimens in water; $\rho_{w}$: density of water.

**Fig. 5:**
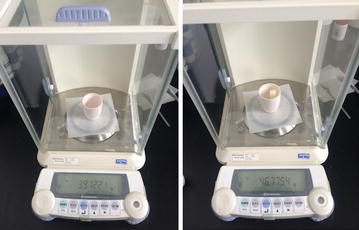
Mass measurement for density test

The mass measurement for density test is as shown in Fig. 5.

### Glass fiber content

According to GB/T 2577-2005, resin content of R glass fiber reinforced DWZ epoxy resin composite was tested.

- Resin mass and volume content percent can be calculated by using formulas (6) and (7):

6 $$M_{r} = \frac{{M_{22} - M_{3} }}{{M_{22} - M_{11} }} \times 100$$

7 $$V_{r} = \frac{{M{}_{r} \times \rho_{c} }}{{\rho_{a} }} \times 100$$

In formulas (6) and (7), M_r_: resin mass content, %; M_11_: mass of pot; M_22_: mass of pot and specimens before roasting; M_3_: mass of pot and remaining fiber after roasting; V_r_: resin volume content, %; $\rho_{c}$: density of R glass fiber reinforced DWZ epoxy resin composite specimens; $\rho_{r}$: density of resin adhesive.

(b) Glass fiber mass and volume content

8 $$M_{g} = \frac{{M_{4} }}{{M_{22} - M_{11} }} \times 100$$

9 $$V_{g} = \frac{{M{}_{g} \times \rho_{c} }}{{\rho_{g} }} \times 100$$

In formulas (8) and (9), M_4_: mass of remaining glass fiber after roasting; M_g_: glass fiber mass content, %; V_g_: glass fiber volume content, %; $\rho_{c}$: density of R glass fiber reinforced DWZ epoxy resin composite specimens; $\rho_{g}$: density of glass fiber.

(c) Void volume content

Void volume content percent can be calculated by using formula (10):

10 $$V_{f} = (1 - V_{r} - V_{g} ) \times 100$$

In formulas (10), V_f_: void volume content, %.

### Thermal shrinkage rate test

Thermal shrinkage rate was measured in the axial, round, and radial directions. The measurements were made using a ‘Resistance Piece Dilatometer’. Resistance Piece Dilatometer transmitted the shrinkage of the specimens at low temperature to a linear variable differential transformer (LVDT) operating at ambient temperature, the principia of thermal shrinkage rate test is as shown in formulas (11),

11 $$\frac{\Delta l}{l} = K_{s} \frac{\Delta R}{R}$$

In formulas (11), $\Delta l/l$: strains of strain gauge; $K_{s}$: sensitivity coefficient of strain gauge; $\Delta R/R$: resistance variation of strain gauge.

**Fig. 6:**
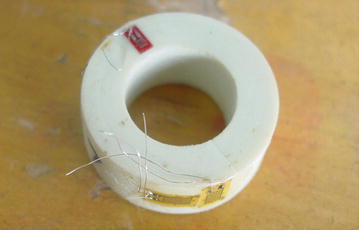
Principia of thermal shrinkage rate test

Figure 6 illustrates the principia of thermal shrinkage rate test. The three strain pieces were assembled on the specimens in radial direction, axial direction and hoop direction respectively. The specimens was allowed to warm-up over a period of 24 h while the temperature and LVDT voltage were recorded.

## Results

### Density measurement

The densities of specimens are as shown in Table 7. For the composite, the density is influenced by the type of glass fiber, geometry of glass fiber, fiber angle and the contents of resin, glass fiber and void. The results indicate the densities of the five specimens is less than 2.000 g/cm^3^. The density of specimen wound by glass fiber tape is lower, which corresponds to higher resin content.

**Table 7:** Testing results of composite density (unit: g)

No.	M_1_ (in air)	M_2_ (in water)	ΔM = M_1_ − M_2_	ρ_c_ (g/cm^3^)
1	10.520	5.010	5.510	1.909
2	7.263	3.565	3.698	1.964
3	14.208	5.771	8.437	1.684
4	10.218	4.753	5.465	1.870
5	10.627	5.267	5.360	1.983

Density of resin is 1.22 g/cm^3^

Density of filament01 and tape is 2.54 g/cm^3^

Density of filament02 is 2.65 g/cm^3^

### Glass fiber and resin content measurements

As shown in Table 8, for the specimen wounded by using glass fiber tape, the volume content of void is higher than other specimens wounded by using glass fiber filament. However, void can lead to micro-crack during cool down, higher volume content of void corresponds to lower cryogenic mechanical properties of composite. To reduce the void and obtain high cryogenic mechanical properties of composite, the insulation tube of isolators for superconducting Tokamak should be wounded by glass fiber filament.

**Table 8:** Content measurement of composite by roasting method

No.	Fiber content		Resin content		Void
	Mass%	Vol%	Mass%	Vol%	Vol%
1	73.184	55.12	26.816	43.31	1.57
2	72.023	53.34	27.977	45.35	1.31
3	58.587	38.83	41.413	58.21	2.96
4	70.121	51.75	29.879	46.36	1.89
5	75.139	56.51	24.861	41.17	2.32

### Thermal shrinkage rate measurement

The testing results of thermal shrinkage rate are as shown in Table 9. To develop isolators for superconducting Tokamak, the thermal shrinkage rate of composite and stainless steel should be basically identical.

**Table 9:** Thermal shrinkage rate of insulation tube (units: %)

No.	α at 77 K			α at 4.2 K		
	Axial	Round	Radial	Axial	Round	Radial
1	0.3750	0.2035	0.3698	0.4473	0.2662	0.4205
2	0.2316	0.0985	0.3565	0.3325	0.1073	0.3951
3	0.2643	0.2831	0.5472	0.3902	0.3955	0.6502
4	0.3464	0.1643	0.3801	0.4124	0.2322	0.4132
5	0.3203	0.1093	0.3569	0.3372	0.1650	0.3895

However, stainless steel is isotropic but composite is anisotropic, which leads to different thermal shrinkage rate in different directions. The thermal shrinkage rate of stainless steel from room temperature to 4.2 K is about 0.3 %. Table 9 indicates the thermal shrinkage rates of composites in axial, round and radial directions are different, the results of No. 5 will be used to simulate the mechanical properties of isolators for superconducting Tokamak. To reduce thermal stress comes from thermal shrinkage rates, much attention should be paid on the structural design of composites.

## Conclusion and discussion

In this paper, design and tests of the R glass fiber reinforced DWZ epoxy resin composites for superconducting Tokamak were performed. The conclusion and discussion are as follows:

1. In the DWZ cryogenic epoxy resin system, the epoxy resin is continuous phase and curing agent is dispersed phase, the two-phase structure under cryogenic temperature can resist crack propagation effectively. To develop cryogenic temperature resistant isolators for superconducting Tokamak, the cured DWZ cryogenic epoxy resin system is an available option.
2. The density of the insulation tube wound by using glass fiber tape is lower than that using glass fiber filament winding, which corresponds to the resin content of insulation tube wound by using glass fiber tape is higher. However, higher resin content will lead to high void and bad properties. Therefore, to obtain higher properties of composite made from R glass fiber and DWZ cryogenic epoxy resin system, it is necessary to wind the insulation tube of isolators for superconducting Tokamak by using glass fiber filament and the appropriate winding angle.
3. The mass contents of fiber of the insulation tube wounded by glass fiber filament and glass fiber tape are 70–75 % and almost 59 % respectively. The volume contents of fiber of the insulation tube wounded by glass fiber filament and glass fiber tape are almost 55 % and almost 39 %. Obviously, the mass content and volume content of fiber of the insulation tube wounded by glass fiber tape are lower.
4. For filament winding, the fiber angle and fiber content strongly influence the thermal shrinkage. The thermal shrinkage rate of insulation tube wounded by glass fiber filament in radial direction is lower than that using glass fiber tape.
5. Additional areas, including mechanical properties at cryogenic temperature, thermal conductivity, dielectric property and anti-radiation properties of the cryogenic temperature resistant glass fiber reinforced epoxy resin composites for superconducting Tokamak (Huang et al. 2014; Kumosa et al. 2005a, b; Baldan et al. 2000; Hikita et al. 2011), need to be investigated further.
